# A longitudinal study on relationships of Chinese postgraduates’ ought-to L2 self, self-efficacy and engagement

**DOI:** 10.3389/fpsyg.2026.1936128

**Published:** 2026-10-26

**Authors:** Wei Huo, Shiping Deng, Xuemei Wang

**Affiliations:** 1 School of Foreign Languages, Shanghai Jiaotong University, Shanghai, China; 2 Department of Applied Foreign Language Studies, Nanjing University, Nanjing, China; 3 School of English Studies, Shanghai International Studies University, Shanghai, China

**Keywords:** cross-lagged panel model, L2 engagement, L2 self-efficacy, longitudinal study, ought-to L2 self, postgraduate learners

## Abstract

Ought-to L2 self, self-efficacy, and L2 engagement play a key role in postgraduate students’ academic English learning. However, longitudinal research on the joint development of these three factors in this setting remains limited. This study explored the relationships among ought-to L2 self, self-efficacy, and L2 engagement in 174 Chinese master’s students across one semester. The data were gathered at three stages: early semester (T1), mid-semester (T2), and late semester (T3). Latent growth curve modeling and cross-lagged panel modeling were applied. The growth models indicated no significant mean linear change in ought-to L2 self, whereas latent-basis models yielded significantly negative mean growth factors for self-efficacy and engagement. The observed means of the latter two constructs declined at mid-semester and partly recovered at the final wave. Individual differences in change were evident for ought-to L2 self and engagement. Both ought-to L2 self and engagement demonstrated significant autoregressive stability, indicating continuity in their relative standing. The significant cross-lagged paths showed the following pattern: T1 ought-to L2 self positively predicted T2 engagement but negatively predicted T2 self-efficacy; T1 self-efficacy significantly predicted higher T2 engagement. In the latter half of the semester, T2 self-efficacy and T2 engagement reciprocally predicted each other at T3, showing positive prospective associations in both directions over that interval. These patterns are consistent with reciprocal relationships between capability beliefs and participation, but do not establish causal effects or the internalization of obligations. The findings suggest pairing clear expectations with achievable tasks and progress-focused feedback throughout the semester.

## 1 Introduction

Motivation helps learners begin L2 learning and sustain effort toward long-term goals (Dörnyei, 1998; Dörnyei and Ryan, 2015). For postgraduate students, language learning takes place alongside coursework, research, and preparation for employment. Understanding how their motivation relates to continued L2 engagement can therefore inform English teaching in postgraduate education.

The L2 Motivational Self System (L2MSS; Dörnyei, 2009) provides a framework for examining these motivational processes. It includes the ideal L2 self, the ought-to L2 self, and the L2 learning experience. The ideal L2 self concerns the language user a learner wishes to become, whereas the ought-to L2 self concerns perceived obligations and the avoidance of negative outcomes. Although the ideal L2 self has generally shown stronger associations with intended effort (Al-Hoorie, 2018), this does not make obligation-based motivation irrelevant. Family expectations and educational requirements can be salient for Chinese learners (Taguchi et al., 2009; You and Dörnyei, 2016). Recent Chinese studies, however, report different relationships between ought-to motivation and engagement across learning settings (Li et al., 2025; Liu et al., 2024). Its role among postgraduates therefore requires investigation rather than an assumption that it is uniformly stronger or more beneficial in China.

Whether perceived obligations are accompanied by sustained participation may depend partly on learners’ self-efficacy, or their beliefs about their capacity to complete learning tasks (Bandura, 1997). Engagement may also provide experiences through which learners reassess their capabilities and obligations. Longitudinal research has already examined related processes, including self-efficacy growth, reciprocal links between self-efficacy and flow, and change in future L2 selves (Oh, 2022; Jia et al., 2024; Ye and Hu, 2025). What remains less clear is how ought-to L2 self, self-efficacy, and multidimensional engagement jointly develop and predict one another among Chinese postgraduates. The present study combines latent growth curve modeling (LGCM) to describe trajectories with cross-lagged panel modeling (CLPM) to examine prospective associations across three waves. These analyses address change and temporal prediction, respectively; they do not by themselves establish causal mechanisms.

## 2 Literature review

### 2.1 L2 engagement

L2 engagement refers to learners’ involvement in language-learning activities and is commonly understood through behavioral, emotional, and cognitive dimensions (Fredricks et al., 2004; Hiver et al., 2024). Behavioral engagement includes participation, attention, and sustained effort. Emotional engagement concerns learners’ affective involvement in learning, including interest and enjoyment. Cognitive engagement involves investment in understanding and the use of learning strategies. These dimensions describe how learners participate, feel, and think during learning. They are distinguishable from motivation, which concerns the reasons and goals underlying participation, and from self-efficacy, which concerns perceived capability. In this study, engagement is operationalized through the three dimensions represented in the adapted 12-item measure described in Section 3.3.2. The resulting composite represents overall involvement across those dimensions.

Engagement is associated with academic achievement and is responsive to both learner characteristics and instructional conditions (Lei et al., 2018; Mercer and Dörnyei, 2020). In L2 research, teacher support, emotions, grit, and language mindsets have been examined as correlates or statistical predictors of engagement (Sadoughi and Hejazi, 2021, 2023; Lou et al., 2022; Zhong et al., 2024). In the Chinese university EFL context, Tang et al. (2026) reported an indirect association between teacher support and English academic achievement through engagement, with academic buoyancy moderating the support–engagement association. Using qualitative interviews, Shen et al. (2024) illustrated the interconnections among behavioral, emotional, and cognitive engagement in a Chinese online EFL writing class supported by spherical video-based virtual reality (SVVR). These findings broaden understanding of the instructional conditions and learning experiences associated with engagement. However, associations among variables and qualitative accounts of learning experiences do not establish the temporal direction of the relationship between motivation and engagement. For the present study, the relevant question is whether earlier motivational beliefs predict later engagement and whether engagement, in turn, predicts subsequent beliefs.

Much of the survey evidence discussed above is cross-sectional, with engagement treated as an outcome. Such designs cannot distinguish motivation preceding engagement from engagement contributing to later motivation. This limitation does not apply to all engagement research. Li and Li (2022), for example, used pre and post-intervention questionnaires, while Zhai (2025) examined reciprocal relationships among behavioral engagement, perceived support, and positive emotions over three waves. Neither study tested the joint prospective relationships among ought-to L2 self, self-efficacy, and the behavioral-emotional-cognitive engagement composite considered here. Thus, the remaining gap concerns this combination of constructs and the possibility of feedback from engagement, particularly among postgraduate learners.

### 2.2 Self-efficacy

Self-efficacy refers to individuals’ judgments about their capacity to perform a task within a particular context (Bandura, 1997). Learners with stronger efficacy beliefs tend to set challenging goals, use learning strategies, and persist when they encounter difficulties. Learners with lower efficacy may avoid demanding tasks or reduce their effort. Within social cognitive theory, however, self-efficacy is also shaped by experience. Successful task performance and credible feedback can strengthen perceived capability, whereas repeated difficulty may weaken it. This reciprocal account provides a basis for examining self-efficacy both as a predictor and as a possible outcome of engagement.

Previous research has linked self-efficacy to classroom engagement (Bai et al., 2022) and performance in specific language skills (Mills et al., 2006). In the Chinese university EFL context, Wang et al. (2021) identified distinct self-efficacy profiles associated with differences in academic emotions and English proficiency, adding a person-centered perspective to the study of learners’ capability beliefs. Evidence from strategy instruction also suggests that pedagogical support may help strengthen learners’ self-efficacy (Graham, 2007). Longitudinal evidence is also available. Busse and Walter (2013) followed university students learning German and documented changes in self-efficacy alongside changes in learning effort. Oh (2022) examined L2 self-efficacy over 3 years and related its growth to classroom engagement and other learning outcomes. Together, these studies support examining efficacy beliefs in relation to learning involvement and development, while leaving questions about the reciprocal relationship between self-efficacy and engagement unresolved.

Self-efficacy and future L2 selves also need to be distinguished theoretically. Self-efficacy concerns whether a learner believes a task can be accomplished; a future self-guide concerns the standard that the learner hopes or feels obliged to attain. A learner can therefore report strong obligations but little confidence in meeting them. Conversely, a confident learner may meet requirements without relying heavily on others’ expectations. The relevance of this distinction lies in how capability beliefs and obligation-based standards guide participation and are revised through experience. Section 2.5 develops these connections using social cognitive theory and regulatory focus theory.

### 2.3 Ought-to L2 self

Dörnyei’s (2009) L2MSS draws on possible selves theory (Markus and Nurius, 1986) and self-discrepancy theory (Higgins, 1987). Its ideal and ought-to self-guides can also be interpreted through regulatory focus theory (Higgins, 1998). Promotion concerns aspirations and advancement, whereas prevention concerns responsibilities and avoiding undesirable outcomes. Papi et al. (2019) further distinguish the standpoint of each self-guide: ought L2 self/own concerns learners’ own perceived duties, while ought L2 self/other concerns obligations attributed to significant others. Standpoint and regulatory focus are separate distinctions. In particular, an own-standpoint obligation should not automatically be equated with intrinsically motivated or fully autonomous learning.

Evidence for the ought-to L2 self varies with the outcome being studied and the way the construct is measured. Al-Hoorie (2018) meta-analysis found a positive association with intended effort but little association with objectively measured achievement. Intended effort is not equivalent to enacted, multidimensional engagement, so these results cannot be transferred directly to the present outcome. Papi et al. (2019) also showed that distinguishing own and other standpoints changes how the motivational role of ought self-guides is understood. These findings favor a more specific question about which obligations relate to which learning processes, rather than a general conclusion that ought-to motivation is either effective or ineffective.

Recent evidence from China does not support a uniform cultural explanation. Li et al. (2025) found a positive direct association between ought-to L2 self and engagement among adult learners in online university courses. In contrast, Liu et al. (2024) found no statistically significant direct association between ought-to L2 self and AI-mediated informal English learning among Chinese undergraduates. Zhou and Papi (2023) semester-long study of Chinese undergraduates found that ideal self-guides, rather than ought self-guides, predicted the speech outcomes examined. These studies differ in participants, learning settings, measures, and outcomes. Their differences therefore indicate context sensitivity, not evidence that the motivational role of Chinese culture itself has changed over time.

For Chinese postgraduates, expectations from teachers, families, and degree programs may remain salient, but their motivational consequences should not be assumed to be uniformly positive. The present study focuses on perceived obligations in a compulsory English course and relates them to capability beliefs and engagement. It does not measure individual collectivism or compare cultures. Cultural context therefore informs the research rationale without serving as a tested explanation of the observed associations. Interpretation also needs to respect the distinction between externally referenced obligations and personally endorsed duties, as discussed in Section 3.3.3.

### 2.4 Empirical relationships and existing longitudinal evidence

Evidence on the relationships among the three constructs comes from partially overlapping research traditions. For self-efficacy and engagement, Bai et al. (2022) cross-sectional study established a positive association but could not test feedback from participation to later confidence. Busse and Walter (2013) documented declines in learning effort and in self-efficacy for listening and speaking during the first year of university language study. Oh (2022) linked three-year growth in efficacy to classroom engagement and other outcomes. These longitudinal studies move beyond a single survey, but neither tests the full reciprocal model of ought-to L2 self, efficacy, and engagement examined here.

Recent Chinese studies provide contrasting evidence on the direct and indirect associations of ought-to L2 self with online classroom engagement and AI-mediated informal English learning (Li et al., 2025; Liu et al., 2024). Although their settings and outcome measures differ, the survey components of these studies do not establish whether obligations precede participation or participation changes perceived obligations. Kormos et al. (2011), drawing on a survey of 518 learners in Chile, proposed an integrated account of goals, attitudes, and self-related beliefs. Efficacy-related content was embedded in their ideal-self measure and discussed in the proposed framework, rather than tested as a separate self-efficacy construct. This study therefore offers a conceptual connection, but does not establish temporal ordering between self-efficacy and ought-to L2 self. Evidence for an ideal-self relationship cannot simply be assumed to apply to an ought-to relationship.

Some studies already test reciprocal relationships over time. Jia et al. (2024) followed 113 EFL learners weekly for eight weeks. Self-efficacy consistently predicted subsequent flow, while reverse associations occurred at only some occasions. This is relevant evidence of temporally uneven reciprocity, although flow is not equivalent to behavioral-emotional-cognitive engagement and ought-to L2 self was not included. Zhai (2025) three-wave study likewise examines reciprocal links involving behavioral engagement, but with perceived support and positive emotions rather than the two self-related constructs studied here. Thus, reciprocal engagement research exists; the unresolved issue concerns the composition and scope of the present model.

Longitudinal work on L2 selves also warrants explicit recognition. Zhou and Papi (2023) examined how future self-guides predicted speech development over a semester among 83 Chinese undergraduates. Ye and Hu (2025) used three assessments with 391 Chinese secondary school students to examine motivational interventions and found sustained changes in ideal and ought-to L2 selves. These designs address speech development or intervention-related change, rather than reciprocal paths among obligations, efficacy, and engagement. They also concern different educational stages from postgraduate study.

Taken together, the literature supports studying these constructs as related but distinct. It does not justify claiming that longitudinal or reciprocal research is absent. The present contribution is more specific: repeated measurement of all three constructs in the same postgraduate cohort, attention to both overall trajectories and prospective associations, and examination of engagement as a possible antecedent of later motivational beliefs. This design also permits a comparison of the patterns observed across two equal intervals, while reserving claims about differences in effect size for formal statistical tests.

### 2.5 An integrated theoretical framework

The framework combines three complementary accounts. The L2MSS identifies the perceived standards that orient language learning; regulatory focus theory explains how aspirations and obligations can direct goal pursuit; and social cognitive theory explains how perceived capability relates to action and is revised through experience (Dörnyei, 2009; Higgins, 1998; Bandura, 1997). Engagement represents learners’ behavioral, emotional, and cognitive involvement in this process. No construct is treated as a substitute for the others, and their integration does not require a single sequence from obligation to efficacy to engagement.

Ought-to standards may encourage learners to participate in order to meet requirements or avoid disappointing significant others. Yet the same standards can make a shortfall between perceived capability and expected performance more salient. Jiang and Papi (2022) found that own and other-standpoint ought self-guides were positively related to anxiety among Chinese EFL learners. This is evidence about anxiety, not proof that obligation lowers efficacy. It nevertheless helps motivate the theoretical possibility that obligation may coincide with effort while being less favorable to confidence. Prevention should not be equated with disengagement: Papi et al. (2025) found that a prevention-oriented task elicited more cognitive engagement than a promotion-oriented task. Effects can therefore differ across dimensions of involvement.

Self-efficacy may influence how manageable an obligation appears. Stronger efficacy could support efforts to meet a valued duty, or reduce the salience of external pressure when learners feel able to meet requirements. Conversely, demanding standards may strengthen confidence when successfully attained or weaken it when experienced as unattainable. These alternatives motivate testing both prospective directions between efficacy and ought-to L2 self without imposing a uniform positive or negative relationship. They do not imply that efficacy is itself a promotion orientation, or that a statistical interaction between efficacy and regulatory focus has been tested.

For efficacy and engagement, social cognitive theory provides a clearer basis for anticipating positive reciprocal associations. Capability beliefs can support persistence and strategic investment, while successful participation can provide mastery information that strengthens subsequent efficacy. The reverse pathway depends on the quality and interpretation of experience: participation without success need not build confidence. Engagement may also alter the salience of obligations by helping learners meet expectations, or by exposing unmet requirements. The direction of feedback to ought-to L2 self is therefore left open.

The empirical model examines prospective associations implied by this framework, not the mechanisms themselves. Regulatory focus, anxiety, mastery experiences, and internalization were not measured as separate variables. They are used to explain plausible connections and identify directions for future work. The CLPM includes reciprocal paths among the three measured composites, whereas the separate LGCMs describe their developmental forms. This distinction links the theoretical rationale to the actual analyses without claiming mediation, moderation, or causal identification.

### 2.6 The current study

The preceding review identifies a specific gap: existing studies have established change in motivational beliefs and reciprocal associations in related domains, but provide limited evidence on the joint three-wave relationships among ought-to L2 self, self-efficacy, and behavioral-emotional-cognitive engagement in Chinese postgraduate English learning. Studying the three constructs together can show whether an association between two remains evident when the third is included. It also permits examination of engagement as both a predicted outcome and a predictor of subsequent beliefs.

The study follows the same postgraduate learners at Weeks 4, 12, and 20 of a semester. Separate LGCMs describe each construct’s trajectory and individual differences in change. An observed-score CLPM estimates prospective paths in both directions conditional on each construct’s prior level and the other lagged predictors. The two eight-week intervals allow patterns at different stages of the course to be examined. Conventional CLPM paths nevertheless combine stable between-person differences with occasion-specific variation and should not be interpreted as isolated within-person causal effects (Hamaker et al., 2015). The following questions therefore concern developmental patterns and prospective associations rather than causal mediation.

Guided by these aims, the present study addresses the following research questions:

*RQ1*: How do Chinese postgraduate learners’ Ought-to L2 Self, L2 self-efficacy, and L2 engagement change over time?

*RQ2*: What prospective associations, including reciprocal associations, are observed among these constructs across the two measurement intervals?

## 3 Methods

### 3.1 Participants

The participants in this study were 196 first-year Chinese postgraduate students from a comprehensive university in North China. All were non-English majors who were taking a compulsory Comprehensive English course during their first academic year. Of these students, 22 (11.2%) did not complete all three measurement waves and were therefore excluded from the longitudinal analyses. The final analytic sample comprised 174 students. In the final analytic sample, the participants were drawn from five schools: Mechanical Engineering (*n* = 39), Mathematics (*n* = 40), Materials Science and Engineering (*n* = 46), Humanities and Social Sciences (*n* = 22), and Economics and Management (*n* = 27). Of these participants, 98 were female (56.3%) and 76 were male (43.7%). Most of the participants were between 22 and 25 years old (*n* = 164); a small number were over 26 years old (*n* = 8) or under 21 years old (*n* = 2). All the participants had completed their undergraduate studies in different fields before enrolling in the master’s program.

At the university where this study took place, postgraduate students are required to complete a one-year English course, and passing the final exams in both semesters is necessary for graduation. English classes were organised by department, and aimed to develop both academic English proficiency and communication skills. Convenience sampling was used to collect the data at three measurement occasions during the academic semester, corresponding to the beginning, middle and end of the semester.

### 3.2 Data collection

Two associate professors who taught the postgraduate Comprehensive English course assisted with data collection. One held a bachelor’s degree and had 18 years of teaching experience; the other held a master’s degree and had 13 years of teaching experience. They taught students from two and three departments, respectively.

Data were collected during the first semester of the 2025–2026 academic year. T1 took place in Week 4, T2 in Week 12, and T3 in Week 20. The measurement occasions were therefore separated by 8 weeks.

At all the measurement points, the participants completed an online questionnaire during their regular class hours, and it took about 15 min. Participation was voluntary, and students were informed that they could leave the study at any point without any negative consequence. To observe changes over time, the same group of students was surveyed in all three rounds, and their university identification numbers were used to match the answers accurately at the three collection times.

### 3.3 Measures

#### 3.3.1 Self-efficacy

English-learning self-efficacy was measured using five items adapted from Ueki and Takeuchi (2012), for example, “I am sure that I will be able to master English skills.” Responses were recorded on a five-point Likert scale from 1 (strongly disagree) to 5 (strongly agree). Higher mean scores indicated stronger perceived capability to achieve English-learning goals.

#### 3.3.2 L2 engagement

Guided by the behavioral-emotional-cognitive framework outlined in Section 2.1 (Fredricks et al., 2004; Hiver et al., 2024), L2 engagement was assessed using 12 items selected and adapted from the corresponding three dimensions of Li and Li’s (2022) scale, with four items per dimension. Items measuring social engagement were not included, consistent with the conceptual scope of the present study. Behavioral items concerned sustained effort and classroom participation; emotional items concerned feelings toward language learning; and cognitive items concerned mental investment and learning strategies. All items were rated on a five-point Likert scale ranging from 1 (strongly disagree) to 5 (strongly agree). Mean scores across the 12 items were used to represent overall L2 engagement in the longitudinal analyses, so the reported paths concern overall engagement rather than separate effects for each dimension.

#### 3.3.3 Ought-to L2 self

The ought-to L2 self was assessed using a four-item measure adapted from Papi et al. (2019), targeting learners’ perceived duties, obligations, and expectations concerning English learning. All items were rated on a five-point Likert scale ranging from 1 (strongly disagree) to 5 (strongly agree). Mean scores across the four items were used in the longitudinal analyses. The adapted measure was analyzed as an overall ought-to L2 self composite, without separate estimates for own and other-standpoint self-guides. The findings therefore concern the composite measure and should not be attributed exclusively to external expectations or interpreted as evidence of their internalization. Internal consistency estimates are reported in Table 1.

**Table 1:** Reliability, convergent validity, and within-wave composite-score correlations.

Variables	Cronbach’s α	Factor loading range	CR	AVE	1	2	3
1. Ought-to L2 self-T1	0.926	0.84–0.93	0.928	0.762	0.**873**		
2. Self-efficacy-T1	0.914	0.75–0.88	0.914	0.682	−0.160*	0.**826**	
3. Engagement-T1	0.909	0.80–0.95	0.891	0.733	0.288**	0.220**	0.**856**
1. Ought-to L2 self-T2	0.922	0.84–0.89	0.922	0.749	0.**865**		
2. Self-efficacy-T2	0.925	0.80–0.88	0.927	0.717	−0.094	0.**847**	
3. Engagement-T2	0.977	0.89–0.97	0.954	0.873	−0.055	0.079	0.**934**
1. Ought-to L2 self-T3	0.943	0.87–0.92	0.943	0.806	0.**898**		
2. Self-efficacy-T3	0.903	0.77–0.86	0.905	0.657	−0.073	0.**811**	
3. Engagement-T3	0.947	0.86–0.98	0.949	0.861	−0.172*	0.254**	0.**928**

* *p* < 0.05; ** *p* < 0.01. Bold diagonal values are √AVE; off-diagonal values are Pearson correlations between observed composite scores (*N* = 174), also reported in Table 2. For engagement, *α* is based on 12 items, whereas CR is based on the three standardized second-order loadings and their residual variances.

### 3.4 Data analyses

Before data collection, an *a priori* Monte Carlo power analysis was conducted to plan the sample size for the primary three-wave observed-score cross-lagged panel model (CLPM). Based on 5,000 simulated datasets, a two-tailed *α* of 0.05, a target power of 0.80, and a pre-specified focal cross-lagged effect of *β* = 0.25, a complete-case sample of 170 was estimated to yield power of 0.916. This target assumed 15% attrition from the maximum feasible baseline recruitment of 200 students. The final complete-case sample was 174, exceeding the planned target. This power estimate applies to the pre-specified focal path under the simulation assumptions and does not establish adequate power for every parameter or model in the study.

The measurement quality of the study variables was examined at each of the three waves using confirmatory factor analysis (CFA). Engagement was represented by three first-order factors—behavioral, emotional, and cognitive engagement—and a second-order engagement factor. Cronbach’s α was calculated from the items used to form each observed composite. For engagement, α was based on all 12 items, whereas composite reliability (CR) was calculated from the three standardized second-order loadings and their residual variances. The engagement CR therefore concerns the second-order measurement structure; it is not another estimate of the reliability of the 12-item mean score. Model-based reliability estimates depend on the specified measurement model (Raykov, 1997). The two coefficients should consequently be interpreted at their respective levels rather than ranked as interchangeable estimates. Model fit was evaluated using *χ*^2^/df, the comparative fit index (CFI), the Tucker–Lewis index (TLI), the root mean square error of approximation (RMSEA), and the standardized root mean square residual (SRMR). CFI and TLI values around 0.90 or higher and RMSEA values below 0.08 were used as guides to acceptable fit, with values closer to 0.95 for CFI/TLI and 0.05 for RMSEA indicating better fit; these guidelines were considered jointly rather than as universal rules (Hu and Bentler, 1999; Marsh et al., 1988). Longitudinal measurement invariance was examined through configural, metric, and scalar models, considering *χ*^2^ difference tests alongside changes in CFI, RMSEA, and SRMR (Chen, 2007).

Separate latent growth curve models (LGCMs) were estimated to examine average trajectories and between-person differences in change (Kline, 2016). Preliminary inspection of the observed means showed a decline from T1 to T2 followed by recovery at T3 for self-efficacy and L2 engagement. A common linear specification would impose a constant rate of change across the two equal intervals and could obscure these patterns. The parallel-process linear model was therefore not retained as the final specification. A linear LGCM was estimated for ought-to L2 self, whereas latent-basis LGCMs were estimated for self-efficacy and engagement to allow different amounts of change across the two intervals. Because the models were estimated separately, cross-process intercept and growth-factor covariances were neither estimated nor interpreted.

An observed-score cross-lagged panel model (CLPM) was used to examine prospective associations among ought-to L2 self, self-efficacy, and L2 engagement across the three waves (Figure 1). Each later score was regressed on its own prior score and the prior scores of the other two constructs. The final analytic sample contained 174 participants with complete data at all three waves. Descriptive statistics were calculated for the nine repeated composite scores. Reported skewness and kurtosis values were within ±2, indicating no marked univariate departures from normality; these checks do not establish multivariate normality. Models were estimated using maximum likelihood with robust standard errors. For the CLPM, bias-corrected 95% bootstrap confidence intervals and two-sided bootstrap *p* values for unstandardized coefficients were obtained from 5,000 resamples. Composite mean scores from the adapted measures were used as observed variables. An item-level latent-variable CLPM would explicitly account for measurement error but would require a more complex measurement structure and substantially more parameters. Given the final sample size, the observed-score model provided a more parsimonious analysis. The measurement analyses informed this choice, but satisfactory reliability and approximate measurement invariance do not eliminate measurement error in the composite scores.

**Figure 1:**
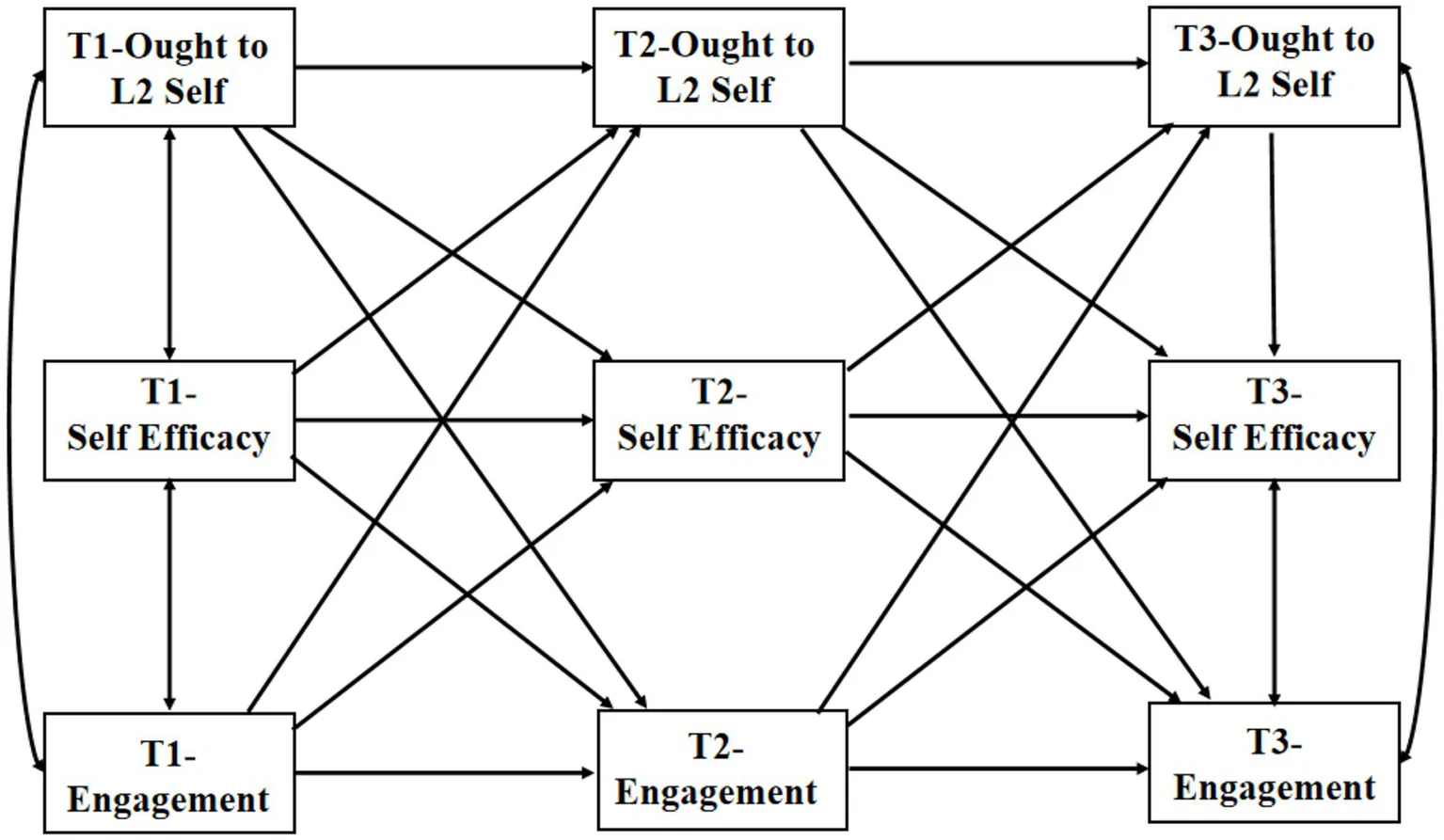
Hypothesized cross-lagged panel model of the dynamic interplay among L2 engagement, ought-to self and self-efficacy across three time points (T1–T3).

## 4 Results

Table 2 presents the means, standard deviations, and Pearson correlations (r) for all nine repeated composite scores in the final analytic sample (*N = 174*). The measurement and longitudinal model results follow.

**Table 2:** Descriptive statistics and correlation of the study variables.

Variable	1	2	3	4	5	6	7	8	9
1. Ought-to self T1	1								
2. Ought-to self T2	0.235**	1							
3. Ought-to self T3	0.124	0.237**	1						
4. Self-efficacy T1	−0.160*	−0.155*	−0.167*	1					
5. Self-efficacy T2	−0.240**	−0.094	−0.325**	0.156*	1				
6. Self-efficacy T3	0.142	0.030	−0.073	0.163*	0.400**	1			
7. Engagement T1	0.288**	−0.084	−0.054	0.220**	0.075	0.077	1		
8. Engagement T2	0.180*	−0.055	−0.091	0.260**	0.079	0.232**	0.265**	1	
9. Engagement T3	0.051	0.025	−0.172*	0.187*	0.348**	0.254**	0.238**	0.605**	1
Mean	2.889	2.832	3.007	3.161	2.676	3.031	3.002	2.591	2.940
Standard deviation	0.927	0.867	0.887	0.976	0.997	0.844	0.897	1.563	1.207

*p < 0.05; *^*^p < 0.01.

### 4.1 Measurement model and longitudinal measurement invariance

The single-wave CFA results indicated generally acceptable fit across the three constructs (Table 3). Standardized factor loadings ranged from 0.75 to 0.98. Internal consistency and convergent validity were also satisfactory: Cronbach’s α ranged from 0.903 to 0.947, CR ranged from 0.891 to 0.954, and AVE ranged from 0.657 to 0.873 (Table 1).

**Table 3:** Fit indices for the single-wave confirmatory factor analysis models (*N* = 174).

Model	*χ^2^*/df	RMSEA	GFI	AGFI	CFI	TLI
Engagement-T1	2.070	0.078	0.934	0.901	0.943	0.926
Engagement-T2	1.359	0.046	0.935	0.900	0.988	0.985
Engagement-T3	1.659	0.062	0.928	0.889	0.978	0.972
Self-efficacy-T1	1.145	0.029	0.990	0.969	0.988	0.979
Self-efficacy-T2	1.226	0.036	0.989	0.967	0.996	0.990
Self-efficacy-T3	1.177	0.032	0.996	0.987	0.992	0.990
Ought-to L2 self-T1	1.769	0.067	0.990	0.950	0.997	0.992
Ought-to L2 self-T2	1.184	0.033	0.974	0.872	0.984	0.953
Ought-to L2 self-T3	1.259	0.039	0.993	0.965	0.999	0.998

The observed composite scores showed weak to moderate interconstruct correlations at each wave, ranging from −0.172 to 0.288. The square root of AVE for each construct exceeded the absolute values of its correlations with the other composites. These comparisons provide descriptive support for separation among the observed scores; the correlations in Table 1 are Pearson correlations between composites, not CFA latent-factor correlations.

Longitudinal measurement invariance was tested across the three waves. The configural model showed acceptable fit, CFI = 0.928, TLI = 0.919, RMSEA = 0.048, and SRMR = 0.050. The metric model did not differ significantly from the configural model, Δ*χ*^2^(32) = 35.98, *p* = 0.288, with negligible changes in approximate fit (ΔCFI = −0.0004, ΔRMSEA = −0.0003, and ΔSRMR = +0.0004). The scalar model differed significantly from the metric model, Δ*χ*^2^(32) = 106.66, *p* < 0.001. Thus, the *χ*^2^ difference test rejected the added scalar constraints. Nevertheless, the changes in the approximate fit indices were small (ΔCFI = −0.0078, ΔRMSEA = +0.0021, and ΔSRMR = −0.0005). Considering these indices jointly (Chen, 2007), the scalar model was retained as an approximate basis for longitudinal comparison, rather than as evidence that exact scalar invariance had been established.

### 4.2 Observing changes over time

LGCMs were estimated to examine change in ought-to L2 self, self-efficacy, and L2 engagement. As shown in Table 4, all three models demonstrated acceptable fit. The linear model for ought-to L2 self and the latent-basis models also fitted the data adequately for self-efficacy and engagement.

**Table 4:** Fit indices of the separate LGCMs (*N* = 174).

Model	*χ*^2^/df	CFI	TLI	RMSEA	SRMR
Ought-to L2 self	1.882	0.951	0.951	0.071	0.033
Self-efficacy	1.735	0.965	0.948	0.065	0.041
Engagement	1.953	0.979	0.968	0.074	0.013

As shown in Table 5, the linear LGCM indicated no statistically significant mean linear change in ought-to L2 self across the semester (M_Growth = 0.12, *p* > 0.05). Self-efficacy and L2 engagement were modeled using latent-basis LGCMs, which allowed change to differ between the two measurement intervals. The mean growth factors were significantly negative for self-efficacy (M_Growth = −0.24, *p* < 0.05) and engagement (M_Growth = −0.20, *p* < 0.05).

**Table 5:** Results of the LGCM of ought-to L2 self, self-efficacy and engagement.

Variable	Functional form	Intercept		Growth		Intercept-Growth covariance
		M	Var	M	Var	
Ought-to Self	Linear	2.85**	0.24**	0.12	0.30**	−0.16
Self-efficacy	Latent-basis	3.20**	0.31**	−0.24*	0.16	−0.09
Engagement	Latent-basis	3.00**	0.16	−0.20*	0.24*	0.15*

M = mean; Var = variance. * *p* < 0.05; ** *p* < 0.01.

The intercept variances were significant for ought-to L2 self (Var_Intercept = 0.24, *p* < 0.01) and self-efficacy (Var_Intercept = 0.31, *p* < 0.01), indicating between-person differences in initial levels. For engagement, the intercept variance was not statistically significant (Var_Intercept = 0.16, *p* > 0.05). Significant growth-factor variances were found for ought-to L2 self (Var_Growth = 0.30, *p* < 0.01) and engagement (Var_Growth = 0.24, *p* < 0.05), indicating that students differed in their patterns of change over the semester. For self-efficacy, the growth-factor variance was not significant (Var_Growth = 0.16, *p* > 0.05), although the mean growth factor was significantly negative. Thus, the model detected significant mean change in self-efficacy without detecting significant between-person differences in that change.

The intercept–growth covariances were not statistically significant for ought-to L2 self (Cov = −0.16, *p* > 0.05) or self-efficacy (Cov = −0.09, *p* > 0.05), providing no significant evidence of an association between initial levels and subsequent change in these constructs. For engagement, the intercept–growth covariance was positive and significant (Cov = 0.15, *p* < 0.05), indicating that students with higher initial engagement tended to have more positive growth-factor scores.

### 4.3 Prospective associations over time

A cross-lagged panel model (CLPM) was estimated to examine prospective associations among ought-to L2 self, self-efficacy, and L2 engagement across the three measurement waves. The model included autoregressive and cross-lagged paths, allowing each construct at a later wave to be predicted by its own prior level and the prior levels of the other two constructs. The model showed acceptable fit to the data (*χ*^2^/df = 1.482, *p* = 0.102, CFI = 0.970, TLI = 0.927, SRMR = 0.045 and RMSEA = 0.053). Table 6 presents standardized coefficients (*β*), unstandardized coefficients (B), standard errors for B, bias-corrected 95% bootstrap confidence intervals for B, and two-sided bootstrap *p* values for all autoregressive and cross-lagged paths.

**Table 6:** Unstandardized and standardized autoregressive and cross-lagged path coefficients.

Path	*β*	B	SE(B)	BC 95% CI for B	*p*
Engagement T1 → Engagement T2	0.16	0.28	0.13	[0.02, 0.54]	0.04
Self-efficacy T1 → Engagement T2	0.25	0.41	0.12	[0.17, 0.64]	< 0.01
Ought-to L2 self T1 → Engagement T2	0.17	0.29	0.13	[0.05, 0.55]	0.02
Engagement T1 → Ought-to L2 self T2	−0.14	−0.14	0.08	[−0.29, 0.01]	0.07
Self-efficacy T1 → Ought-to L2 self T2	−0.08	−0.07	0.07	[−0.21, 0.06]	0.28
Ought-to L2 self T1 → Ought-to L2 self T2	0.26	0.25	0.07	[0.10, 0.39]	< 0.01
Engagement T1 → self-efficacy T2	0.13	0.15	0.09	[−0.02, 0.32]	0.09
Self-efficacy T1 → self-efficacy T2	0.08	0.09	0.08	[−0.07, 0.24]	0.27
Ought-to L2 self T1 → self-efficacy T2	−0.26	−0.28	0.08	[−0.45, −0.12]	< 0.01
Engagement T2 → Engagement T3	0.59	0.45	0.04	[0.37, 0.54]	< 0.01
Ought-to L2 self T2 → Engagement T3	0.09	0.12	0.08	[−0.03, 0.27]	0.12
Self-efficacy T2 → Engagement T3	0.31	0.38	0.07	[0.24, 0.51]	< 0.01
Engagement T2 → Ought-to L2 self T3	−0.06	−0.03	0.04	[−0.11, 0.05]	0.43
Ought-to L2 self T2 → Ought-to L2 self T3	0.21	0.21	0.07	[0.07, 0.35]	< 0.01
Self-efficacy T2 → Ought-to L2 self T3	−0.30	−0.27	0.06	[−0.39, −0.15]	< 0.01
Engagement T2 → self-efficacy T3	0.21	0.11	0.04	[0.04, 0.18]	< 0.01
Ought-to L2 self T2 → self-efficacy T3	0.08	0.08	0.07	[−0.05, 0.21]	0.25
Self-efficacy T2 → self-efficacy T3	0.39	0.33	0.06	[0.22, 0.44]	< 0.01

*β* = standardized coefficient; B = unstandardized coefficient; SE(B) = standard error of B; BC = bias-corrected. The 95% confidence intervals and two-sided *p* values for B were obtained from 5,000 bootstrap resamples.

Ought-to L2 self showed significant positive autoregressive paths from T1 to T2 (*β* = 0.26, *p* < 0.01) and from T2 to T3 (*β* = 0.21, *p* < 0.01). L2 engagement also showed significant positive autoregressive paths across both intervals (T1 to T2: *β* = 0.16, *p* = 0.04; T2 to T3: *β* = 0.59, *p* < 0.01). For self-efficacy, the autoregressive path from T1 to T2 was not statistically significant (*β* = 0.08, *p* = 0.27), whereas the path from T2 to T3 was positive and significant (*β* = 0.39, *p* < 0.01). These coefficients describe the prediction of later scores by earlier scores of the same construct, conditional on the other lagged predictors in the model.

From T1 to T2, ought-to L2 self positively predicted subsequent engagement (*β* = 0.17, *p* = 0.02) and negatively predicted subsequent self-efficacy (*β* = −0.26, *p* < 0.01). Self-efficacy at T1 also positively predicted engagement at T2 (*β* = 0.25, *p* < 0.01). The paths from T1 engagement to T2 ought-to L2 self and self-efficacy, and from T1 self-efficacy to T2 ought-to L2 self, were not statistically significant (all *p* values > 0.05).

From T2 to T3, self-efficacy positively predicted subsequent engagement (*β* = 0.31, *p* < 0.01) and negatively predicted subsequent ought-to L2 self (*β* = −0.30, *p* < 0.01). Engagement at T2 positively predicted self-efficacy at T3 (*β* = 0.21, *p* < 0.01), indicating reciprocal positive prospective associations between self-efficacy and engagement over this interval. The paths from T2 ought-to L2 self to T3 engagement and self-efficacy, and from T2 engagement to T3 ought-to L2 self, were not statistically significant (all *p* values > 0.05). Self-efficacy predicted later engagement in both intervals, whereas the reverse path reached significance only from T2 to T3. This difference in statistical significance does not establish a difference between the interval-specific coefficients. Indirect effects through T2 variables were not tested.

## 5 Discussion

This study tracked Chinese postgraduate learners across one academic semester to examine change and prospective associations among ought-to L2 self, self-efficacy, and L2 engagement. Separate LGCMs described average trajectories and between-person variability within each construct. A CLPM then examined the prediction of later scores by earlier scores across constructs.

### 5.1 Overall growth of variables

The three constructs showed different patterns of change. Ought-to L2 self showed no significant mean linear change. For self-efficacy and engagement, the observed means declined from T1 to T2 and partly recovered at T3 (Table 2), while the latent-basis models yielded significantly negative mean growth factors (Table 5). The growth factors are defined by the models’ time loadings and should be distinguished from the raw differences between the observed T1 and T3 means. Relatively stable average obligations can therefore coexist with changing confidence and participation. Course requirements may help maintain expectations, whereas learning experiences and workload may relate differently to the other constructs. These contextual explanations remain provisional because the proposed influences were not measured directly.

Significant slope variances for ought-to L2 self and engagement indicate differences between students in their patterns of change. The nonsignificant slope variance for self-efficacy means that the model did not detect reliable variation in change across students; it does not show that self-efficacy itself was unchanged. This distinction matters because a group can show mean change without detectable differences in the amount of change. Social cognitive theory suggests that mastery experiences and feedback may contribute to efficacy development (Bandura, 1997), but the present data do not identify which experiences account for the observed trajectory.

The intercept–growth associations also require interpretation in relation to the fitted growth form. Initial self-efficacy was not significantly associated with its growth factor. For engagement, the positive intercept–growth covariance indicates that higher initial engagement was associated with more positive growth-factor scores. This association concerns the modeled pattern of change and does not by itself establish continuous growth or an inevitable widening gap between learners.

The decline and subsequent recovery in self-efficacy and engagement suggest that support should continue beyond the opening weeks. Low-stakes tasks, scaffolded presentations, and staged writing assignments may provide opportunities for successful performance throughout the course. Specific feedback on progress could help students interpret demanding tasks as manageable. These are pedagogical implications to be evaluated, rather than interventions tested in this study.

Early participation may still be useful for identifying students who need support. Brief discussion tasks and peer review can offer manageable opportunities to become involved. However, the observed associations do not imply that initially less-engaged students cannot catch up or that a particular classroom routine will necessarily alter their trajectory.

The combination of a positive early association between obligation and engagement and a negative association between obligation and self-efficacy also suggests care in communicating expectations. Teachers can make requirements clear while explaining their academic purposes and providing achievable steps toward meeting them. This approach acknowledges obligations without assuming that greater pressure will improve both participation and confidence.

### 5.2 Autoregressive effects of variables

The positive autoregressive paths for ought-to L2 self indicate continuity in students’ relative standing across the semester, conditional on the other variables in the model. Stable course requirements and expectations from significant others offer a plausible context for this continuity. However, autoregressive stability does not mean that each student’s level remained unchanged, and it does not identify which social expectations were influential. The significant variation in growth further indicates that obligation-related trajectories differed between students.

Self-efficacy showed a non-significant autoregressive path from T1 to T2 and a significant positive path from T2 to T3. One possibility is that students revised their initial confidence as they encountered postgraduate English tasks and feedback, and that their later judgments drew on more course-specific experience. This interpretation is compatible with social cognitive theory (Bandura, 1997) and longitudinal work on efficacy development (Oh, 2022). Nevertheless, the difference in significance across intervals does not establish a discrete transition from calibration to consolidation; a formal comparison of coefficients would be needed to support that claim.

Engagement showed significant positive autoregressive paths in both intervals. Together with the positive intercept–slope covariance (0.15, *p* < 0.05) and significant slope variance in the LGCM, these findings indicate persistence alongside variation between learners. Zhai (2025) also examined reciprocal relationships involving behavioral engagement over three waves. However, continuity does not by itself identify habit formation, classroom experience, or another process as the mechanism.

These results indicate continuity in engagement, but they do not establish that engagement is more persistent than the other constructs. Such a comparison would require explicit tests of differences between autoregressive coefficients. Stable differences between students and shared instructional conditions may also contribute to the observed continuity.

The autoregressive patterns suggest several considerations for teaching, while leaving the effectiveness of particular strategies open to further investigation.

First, teachers can revisit course expectations and explain how assignments relate to disciplinary communication. This may help students connect perceived obligations with a clearer understanding of the tasks they are expected to complete.

Second, feedback can connect current performance with earlier work, helping students judge progress from specific evidence. Such feedback may be particularly useful when students are still forming course-specific efficacy beliefs.

Third, teachers can offer regular, low-stakes opportunities to contribute, such as prepared discussion questions or rotating group roles. These activities may make participation more accessible to students who are initially less engaged.

### 5.3 Cross-lagged associations between variables

The relationship between ought-to L2 self and self-efficacy was not consistently reciprocal across the semester. T1 ought-to L2 self negatively predicted T2 self-efficacy, whereas T2 self-efficacy negatively predicted T3 ought-to L2 self. The framework in Section 2.5 offers a possible interpretation: obligations can make a gap between expected performance and perceived capability salient, while confidence may change how strongly learners orient themselves toward perceived duties and expectations. Jiang and Papi (2022) provide related evidence linking ought self-guides to anxiety, although anxiety is not self-efficacy. Because this study did not assess anxiety, regulatory focus, or the internalization of obligations, these processes remain explanations to test. The observed paths do not demonstrate that students shifted from externally regulated to autonomous motivation.

Ought-to L2 self positively predicted engagement from T1 to T2, but its T2-to-T3 path was not statistically significant. This pattern is compatible with a role for perceived obligations in early course participation, although the difference between intervals was not formally established. It also adds a longitudinal perspective to the context-sensitive associations reported in recent Chinese research (Li et al., 2025; Liu et al., 2024). Neither the present results nor those cross-sectional comparisons establish that obligation inevitably loses its motivational value. Its relationship with participation may depend on the demands of the setting and the aspects of engagement measured.

Self-efficacy positively predicted subsequent engagement in both intervals. This finding is consistent with the social cognitive account that perceived capability supports effort and persistence (Bandura, 1997). It also complements the associations reported by Bai et al. (2022) and the longitudinal evidence reviewed in Section 2.4. Because the engagement score combines behavioral, emotional, and cognitive involvement, the present paths cannot identify which dimension was most closely associated with efficacy beliefs.

Engagement at T2 also positively predicted self-efficacy at T3, providing evidence of prospective associations in both directions over the second interval. Jia et al. (2024) similarly reported that the reverse paths from flow to efficacy were evident at some, but not all, measurement occasions. Flow is a distinct construct, so the present findings extend this temporal question to a broader engagement composite without treating the two measures as interchangeable.

Successful participation and feedback are plausible sources of later efficacy beliefs. However, engagement does not necessarily produce success, and neither mastery experiences nor feedback were measured as mediators here. The findings are therefore consistent with reciprocal development, but do not establish the mechanism, the duration required for it to occur, or an increase in reciprocity over time. Denser measurement and explicit assessment of learning experiences would help distinguish these explanations.

At T2, the contemporaneous correlations among the three composites were small and nonsignificant (ought-to L2 self with self-efficacy: *r* = −0.094; ought-to L2 self with engagement: *r* = −0.055; self-efficacy with engagement: *r* = 0.079). These values contrast descriptively with the wider ranges at T1 (−0.160 to 0.288) and T3 (−0.172 to 0.254), although differences between waves were not formally tested. Mid-semester tasks, feedback, and competing demands could relate differently to obligations, confidence, and participation, but the present study did not measure these occasion-specific influences. A restricted spread of engagement scores is not evident: its T2 standard deviation was larger than at T1 or T3. The small T2 correlations therefore warrant cautious, context-sensitive interpretation. They do not contradict the significant cross-lagged paths, which concern associations across different occasions conditional on prior scores rather than contemporaneous associations at T2.

The cross-lagged findings suggest that teaching should attend to both perceived obligations and opportunities to develop confidence through participation. The following suggestions follow from the theoretical interpretation and require direct evaluation.

First, teachers can invite students to reflect on whose expectations influence their English learning and how those expectations relate to their academic goals. Clear requirements can be paired with achievable tasks and specific feedback. This may help learners assess what is expected without treating every difficulty as evidence of inadequate ability. It does not require assuming that prevention-oriented goals are inherently harmful or that all learners should adopt a promotion focus.

Second, tasks can include meaningful choices, such as selecting a disciplinary topic or choosing between an oral presentation and a written commentary. These options may support personal relevance while retaining clear assessment standards. Their value should not be justified by a claim that the present study demonstrated internalization, which was not measured.

Third, scaffolded tasks and progress-focused feedback can be provided throughout the semester. Early tasks can offer manageable opportunities for participation; later feedback can connect current performance with earlier work. For example, a teacher might point out how a student’s use of evidence has improved across successive drafts. This provides concrete information for efficacy judgments without prescribing a fixed motivational sequence that the present data cannot establish.

## 6 Conclusion

Going beyond cross-sectional descriptions of L2 motivation, the present study combined LGCM and CLPM analysis to trace how L2 ought-to self, self-efficacy, and engagement change and prospectively predict one another over the course of one semester among Chinese postgraduate students. This longitudinal design provides a more detailed view of how perceived obligations, capability beliefs, and multidimensional engagement are connected over time in a high-stakes context for non-English majors.

The growth analyses detected no significant mean linear change in ought-to L2 self and significantly negative mean growth factors in the latent-basis models of self-efficacy and engagement. The observed means of the latter two constructs declined at T2 and partly recovered at T3. Significant growth-factor variances for ought-to L2 self and engagement further indicated differences between students in their modeled trajectories. These findings distinguish average change from between-person variation in change.

The cross-lagged analyses identified positive prospective associations from self-efficacy to engagement in both intervals and a reverse association from engagement to self-efficacy in the second interval. Early ought-to L2 self predicted higher subsequent engagement but lower self-efficacy, while mid-semester self-efficacy predicted lower later ought-to L2 self. This pattern shows that perceived obligations can relate differently to participation and confidence. It does not establish causal effects, mediated internalization, or statistically verified differences between intervals.

The study contributes evidence on the joint development and prospective relationships of three constructs in a postgraduate EFL setting. Integrating self-guide, regulatory focus, and social cognitive perspectives helps explain why obligations, perceived capability, and participation may be associated in different ways. The findings support further examination of these relationships across learning stages and contexts, including direct tests of the mechanisms proposed here.

Several limitations should be noted. First, the longitudinal design, although stronger than a single-shot survey, covered only three waves within one semester in a single compulsory English course. This design allowed us to capture short-term developments and directional patterns among ought-to L2 self, self-efficacy, and engagement, but it may not fully reflect longer-term motivational restructuring. Postgraduate learners’ obligation, confidence, and engagement may stabilize, erode, or transform across key academic transitions (e.g., thesis writing, internship, job search), which were beyond the present observational window. Future research would benefit from extending the time frame (e.g., tracking students across an academic year or throughout their degree) and increasing the number of measurement occasions, such as incorporating monthly, bi-weekly, or diary-based assessments. A denser time series would enable the detection of non-linear patterns, critical periods, and short-term fluctuations that our three-wave design could not fully capture.

Second, participants were first-year postgraduates in non-English majors taking a compulsory English course at one university in northern China. The findings may not generalize to other institutions, regions, academic stages, or learner groups. Multi-site and comparative studies could examine whether the observed associations also occur among undergraduates, English majors, or students in voluntary language courses. Such comparisons should measure the relevant contextual influences rather than infer them from national or cultural labels. The analyses also excluded 22 students who did not complete all three waves; the results therefore describe completers and may be affected by attrition-related selection.

Third, in terms of analytical modeling, this study used LGCMs and a traditional CLPM to investigate temporal stability and directional associations among the variables. Although the measurement analyses provided support for longitudinal comparisons of the composite scores, the observed-score CLPM did not explicitly account for measurement error, which may have affected the estimated longitudinal associations. While these approaches are appropriate for an initial examination, they do not fully disentangle between-person stability from within-person fluctuations. Future work could apply more advanced frameworks such as random-intercept cross-lagged panel models (RI-CLPM), latent change score models, or dynamic structural equation models, which allow clearer separation of trait-like components and time-specific influences. In addition, experimental or intervention-based designs—for instance, systematically enhancing self-efficacy or modifying how expectations are communicated—would provide stronger evidence for causal mechanisms underlying the observed temporal ordering.

Finally, construct coverage limits interpretation. Engagement was analyzed as a composite of three dimensions, so its prospective associations cannot be attributed to behavioral, emotional, or cognitive involvement separately. The ought-to measure was also modeled as one composite, without separate own and other-standpoint estimates. Future research should report the complete adapted items and examine whether these forms of obligation have distinct relationships with efficacy and engagement. Direct measures of regulatory focus, mastery experiences, and internalization would also be needed to evaluate the proposed mechanisms.

## Data Availability

The original contributions presented in the study are included in the article/supplementary material, further inquiries can be directed to the corresponding author.
